# Exploring Parental Attitudes and Perceptions Regarding Childhood Vaccinations in Greece: A Study Within the Framework of the National Health Examination Survey (EMENO)

**DOI:** 10.7759/cureus.64588

**Published:** 2024-07-15

**Authors:** Alkisti Kotsia, Evmorfia Pechlivanidou, Natasa Kalpourtzi, Georgia Vourli, Vana Papaevangelou, Giota Touloumi, Vasiliki Benetou

**Affiliations:** 1 Department of Hygiene, Epidemiology and Medical Statistics, National and Kapodistrian University of Athens School of Medicine, Athens, GRC; 2 Third Department of Pediatrics, Attikon University Hospital, National and Kapodistrian University of Athens School of Medicine, Athens, GRC

**Keywords:** vaccine-preventable diseases, vaccination hesitancy, skepticism, immunizations, perceptions

## Abstract

Aim: Vaccinations have reduced illnesses and mortality rates globally, yet negative attitudes and uncertainty about them hinder their acceptability and efficacy. The study aims to document Greek parents' immunization perceptions and risk factors.

Methods: Spanning 2014-2017, 447 parents (68% mothers) who participated in the Greek Health Examination Survey EMENO (National Survey of Morbidity and Risk Factors) completed an interview-delivered questionnaire. Attitudes were categorized into three groups: absolutely positive, positive, and negative. Absolutely positive attitudes included positive responses to all five statements in favor of vaccination and negative responses to the two statements against vaccination. Negative attitudes included positive responses to either one or both statements against vaccination and negative responses to all five statements in favor of vaccination.. All other participants were categorized as having a positive attitude. Skepticism towards vaccinations was classified into skeptical and non-skeptical groups based on responses to five statements implying uncertainty or skepticism. Participants were considered skeptical if they provided positive responses to at least three of these statements, and non-skeptical if they had none or up to two positive responses. The statistical analysis accounted for the study design whereas inverse probability weighting was used to adjust for non-response and multiple imputations were employed to impute missing values. The components of parental attitude and vaccine skepticism were identified using weighted multinomial logistic regression and logistic regression, respectively.

Results: In total, 16.6% were classified as having absolutely positive attitudes towards vaccinations whereas 42.1% were skeptical of vaccines. Of all participants, 96.0% agreed that vaccinations are essential for their child’s well-being and adhere to scientific recommendations. However, concerns were also mentioned, with 26.2% worrying about potential adverse effects, and 21.6% believing it is better to acquire immunity through illness rather than vaccination. Positive participants, compared to absolutely positive ones, were more likely to be of Greek origin (adjusted relative rate ratio (aRRR): 3.35; 95% CI: 1.53-7.30) and living in semi-urban areas (aRRR: 4.84; 95% CI: 1.77-13.29). Negative participants, in contrast, were more likely to have higher education (aRRR: 2.98; 95% CI: 1.05-8.44) but also to live in semi-urban areas (aRRR: 6.43; 95% CI: 1.69-24.56). Furthermore, parents of Greek origin had significantly higher odds of being skeptical towards vaccination (adjusted Odds Ratio (aOR): 2.86; 95% CI: 1.36-5.98), while married or cohabiting parents had lower odds of being skeptical compared to single parents (aOR: 0.60; 95% CI: 0.35-1.06).

Conclusions: While parents in this study recognize the importance of childhood immunizations, there is a widespread presence of negative attitudes and skepticism that can have a detrimental impact on vaccination rates.

## Introduction

Despite the great and undisputable success of immunizations in reducing morbidity and mortality from vaccine-preventable diseases over time, \millions of children worldwide do not receive their routine vaccinations for a variety of reasons [1]. As a result, a considerable number of deaths due to vaccine-preventable diseases, especially among children under the age of five, are still occurring, while multiple regions in the world have experienced severe and deadly outbreaks of vaccine-preventable infectious diseases, such as measles, pertussis, and diphtheria [2].

Non-optimal vaccination rates observed globally can be partly attributed to vaccine hesitancy, a complex phenomenon, increasingly recognized during the recent decades, that was characterized by WHO in 2019 as one of the ten most important threats to public health [3]. A wide range of factors have been implicated in the complex decision-making process of being vaccinated mostly related to the general concepts of confidence, complacency, and convenience [4].

In Greece, vaccination is universally recommended and considered mandatory for children in order to attend kindergarten or elementary school. Therefore, vaccines included in the National Vaccination Program (NVP) are provided free of charge to everyone with a national identification number (including refugees), although access to vaccines is provided even to those without a national identification number in children’s hospital outpatient clinics for vaccination. In two relatively recent cross-sectional studies conducted during 2016-2019 among primary school children, one in Patras (3rd largest city in population Greece; Peloponnese) [5], and one in Thessaloniki (2nd largest city in population Greece; Macedonia, North Greece) [6], a high (>90%) vaccine coverage was found for most vaccines (diphtheria, tetanus, acellular pertussis, and inactivated poliovirus vaccine {DTaP-IPV}; Haemophilus influenzae type b vaccine; Hepatitis B vaccine; measles, mumps, and rubella vaccine; varicella {chickenpox} vaccine) although concerns about delayed pneumococcal conjugate vaccine (PCV) vaccination or lack of DTaP booster have been raised. In the former study, a literature review of childhood vaccination coverage in Greece after 2000 was performed; 16 studies covering various areas of Greece were included and the main conclusion was that there was a trend for an increasing over time uptake for most recommended vaccines. Of note, vaccination coverage of certain vulnerable population groups such as immigrant children and Roma was not found optimal according to the 2017 study by Papamichail et al. [7].

Identifying factors associated with reduced children’s vaccine coverage is important, especially for policymakers. Apart from socioeconomic factors [8] parents’ and caregivers’ attitudes and perceptions seem to play an important role in children’s vaccine coverage [4]. In Greece, information on parents’ attitudes and perceptions regarding children's vaccinations, as well as on associated factors, is scarce. Relevant studies have recorded a small, but not negligible, percentage of parents being hesitant towards vaccinations [9]. In a large-scale survey conducted by the Vaccine Confidence Project, among 65,819 individuals across 67 countries in 2015, Greece was rated one of the 10 least confident countries concerning immunizations [10]. On the other hand, in a survey commissioned by the European Commission to compare vaccine confidence rates between 2016 and 2018 in European Union (EU) countries, the state of confidence in vaccines among the public in Greece showed one of the highest improvements [11]. Thus, it has become increasingly important to measure, understand, and address the hesitation and reluctance of parents towards vaccinations in every society and different setting. The aim of this study was to record parental attitudes and perceptions towards vaccinations and to identify the potential determinants of positive or negative attitudes in a large sample of parents residing in Greece. 

## Materials and methods

Population and sampling

A sub-sample of the participants recruited in the context of the Greek Health Examination Survey EMENO (National Survey of Morbidity and Risk Factors) was used for this study. EMENO is a cross-sectional health examination survey conducted during 2013-2016, with the aim to assess the morbidity of chronic diseases and their associated factors in a random sample of adults living in Greece. The study design and methodology have been described in detail by Touloumi et al. [12]. Briefly, EMENO used a multistage, stratified random sampling methodology based on the 2011 Greek census to select a representative sample of the adult population. One of the adults living in the selected households was randomly selected and offered to participate in EMENO. Each participant provided information recorded in the core EMENO questionnaire. The overall response rate in EMENO was 72%.

In the context of this sub-study, each household participating in the EMENO with at least one person <18 years, was eligible for inclusion; during 2014-2017, eligible participants were first contacted by phone to be informed about this study, aiming to investigate various aspects of children’s health, among which immunizations. Those who agreed to participate provided their informed consent. Both the EMENO study as well as this sub-study were approved by the Bioethics & Deontology Committee of the Medical School of National and Kapodistrian University of Athens, Greece (2564/12-11-2014).

Data collection on attitudes and perceptions towards vaccination

After the initial phone contact, trained medical doctors visited households and interviewed one of the parents (the one available or willing to participate) using a structured questionnaire. The questionnaire, previously used and validated in the context of national immunization surveys, comprised 12 statements on perceptions about vaccinations (all statements are shown in detail in Table 1) [13].

**Table 1:** Parent’s responses to the 12 statements concerning attitudes and perceptions toward vaccinations. The data has been represented as N (%).

Statements		Agree (N (%))	Probably agree (N (%))	Probably disagree (N (%))	Disagree (N (%))	Don't know (N (%))
1	Vaccinations are essential for the well-being of my child.	376 (84.1)	53 (11.9)	4 (0.9)	8 (1.8)	6 (1.3)
2	I am deeply concerned about the potential adverse effects of vaccines on my child.	50 (11.2)	67 (15.0)	83 (18.6)	230 (51.5)	17 (3.8)
3	Children should receive immunization with each newly licensed vaccine immediately after approval	91 (20.4)	61 (13.7)	66 (14.8)	202 (45.1)	27 (6.1)
4	Typically, I verify my doctor's recommendations for my child's vaccination through other resources	166 (37.1)	36 (8.1)	18 (4.0)	220 (49.2)	7 (1.6)
5	My child acquiring immunity through illness is superior to their acquiring it through vaccination.	60 (13.4)	37 (8.2)	53 (11.9)	285 (63.8)	12 (2.7)
6	I am perplexed about vaccination due to the divergent viewpoints among doctors.	114 (25.5)	55 (12.3)	37 (8.3)	220 (49.2)	21 (4.7)
7	Presently, I still have intense fear toward vaccines and injections due to a negative encounter I had throughout my childhood.	33 (7.4)	16 (3.6)	12 (2.7)	379 (84.8)	7 (1.6)
8	Increasing the number of vaccines administered to a child significantly improves its overall health.	183 (40.9)	100 (22.4)	47 (10.5)	76 (17.0)	41 (9.2)
9	The state provides minimal information on new vaccines as a cost-saving measure.	200 (44.7)	71 (15.9)	46 (10.3)	45 (10.1)	85 (19.0)
10	I am endeavoring to immunize my child in accordance with scientific recommendations.	375 (83.9)	53 (11.9)	7 (1.6)	9 (2.0)	3 (0.7)
11	Vaccines are entirely benign and incapable of causing significant health issues in children.	103 (23.0)	88 (19.7)	81 (18.1)	113 (25.3)	62 (13.9)
12	Financial gain is the primary motivation behind various measures taken to promote specific new vaccines.	192 (43.0)	109 (24.4)	34 (7.6)	55 (12.3)	57 (12.8)

In particular, five statements were in favor of vaccinations (i.e., questions 1, 3, 8, 10, 11), two statements were against vaccinations (i.e., questions 2 and 5), and five statements implied uncertainty/skepticism toward vaccinations (i.e., questions 4, 6, 7, 9, 12). Briefly, statements attempted to capture perceptions about the importance, safety, or harm of vaccination, confusion concerning existing recommendations, and trust in healthcare providers and their motives. Five possible answers (agree, probably agree, probably disagree, disagree, do not know) were provided for each statement. For the purposes of this study, the answers “agree” and “probably agree” were considered as positive towards the specific statement, while the answers “probably disagree” and “disagree” as negative towards the specific statement.

Data collection on other variables

Socio-demographic data concerning the parent, such as age, educational level, employment, monthly income, area of residence (urban: ≥10,000, semi-urban: 2000-9999, and rural: up to 1999 inhabitants), marital status, nationality, total number of children and insurance of the child, were self-reported and were obtained from the core EMENO questionnaire.

Statistical analysis

Participants were categorized into three groups according to their attitude, towards vaccinations: (a) Absolutely positive (Ap) attitude: participants with positive answers on all the statements in favor of vaccination combined with negative answers on both statements against vaccination (coded as 1), (b) Negative (N) attitude: participants with positive answers on both statements against vaccination, or positive answers on at least one of them combined with negative answers on all statements in favor of vaccination (coded as 3), and (c) Positive (P) attitude: All the other participants were coded as 2. Regarding skepticism, participants were categorized into two groups: a) Skeptical: participants with positive answers on at least three of the five statements that imply uncertainty/skepticism towards vaccination, and b) Non-skeptical: participants with no positive answer or up to two positive answers at the five statements that implied uncertainty/skepticism.

Sampling weights were applied to adjust for the EMENO study design. To adjust for non-response, as not all eligible households participated in this sub-study, the inverse probability weighting method was applied. The weights, derived as reciprocals of response probabilities, were estimated through weighted multivariable logistic regression.

Missing values in the cofactors were imputed using multiple imputations (MI) by the chained equations method (MICE) assuming data were missing at random given observed information. Partially observed cofactors were income, educational level, unemployment, number of kids, nationality, and insurance while gender, area of residence, age, and degree of urbanization were completely observed. Ten imputed datasets were created with a burn-in period of 300 iterations.

Weighted medians and interquartile range (IQR) for continuous variables and weighted percentages for categorical variables were reported. To explore factors associated with parental attitude towards vaccinations, weighted multinomial (as the proportional odds assumption of the ordinal logistic was rejected) logistic regression models were employed; weighted logistic was used to examine factors associated with skepticism.

Statistical analysis was conducted using the software Stata, Version 13.0 (StataCorp, College Station, TX) and R (R Core Team (2023). "R: A Language and Environment for Statistical Computing." R Foundation for Statistical Computing, Vienna, Austria.)

## Results

Of the 6006 participants in the EMENO study, 1522 had at least one child <18 years old living in their household at the time the sub-study was implemented but 45 of them had missing contact information, leaving 1476 eligible for inclusion in this sub-study. Of those, 187 refused to participate, 836 were not approached/found and 453 parents, representing equivalent households, agreed and were included in this sub-study (about 31% response rate overall eligible EMENO participants). The included participants were more likely to be women, with higher educational levels, and living in the Attica region compared to those not included in this sub-study. Six additional participants were excluded from the final analysis as their attitude and skepticism towards vaccination could not be clearly classified due to missing data in some of the relevant statements. Thus, the final study population consisted of 447 parents.

The responses of the 447 participants to each of the 12 statements concerning their attitudes and perceptions toward vaccinations are presented in Table 1. The great majority (96.0%) of the parents agreed and probably agreed that vaccinations are essential for their child’s well-being and that they are trying to vaccinate them in accordance with the scientific recommendations. A total of 63.3% of the parents agreed and probably agreed that increasing the number of vaccines administered to a child significantly improves its overall health, while 42.7% agreed and probably agreed that vaccines were entirely benign and incapable of causing significant health issues. Also, 34.1% agreed and probably agreed that their children should receive immunization with each newly licensed vaccine immediately after approval. On the other hand, 26.2% agreed and probably agreed that they were deeply concerned about the potential adverse effects of vaccines on their child‘s health and 21.6% agreed and probably agreed that it was better for their child to acquire immunity through illness rather than by getting a vaccine. Overall, 16.6% of the parents had an absolutely positive attitude towards vaccinations, 76.3% had a positive attitude, and 7.1% had a negative attitude towards vaccinations. Furthermore, 42.1% of the parents were skeptical toward vaccination. Among them, almost 62.0% declared that they typically, verify their doctor's recommendations for their child's vaccination through other resources, 73.9% agreed and probably agreed that are perplexed about vaccinations due to divergent viewpoints among doctors and 94.7% agreed and probably agreed that financial gain is the primary motivation behind various measures taken to promote specific new vaccines. Among the parents who were absolutely positive or positive towards vaccinations (92.8%), 38.3% were also skeptical, whereas among those who were negative towards vaccinations, 90.6% were also skeptical.

In Table 2 specific characteristics of the parents and their household overall and by attitude and skepticism status towards vaccinations are presented. The interviewed parent was the mother in 68.0% of the study population and the majority were of Greek origin (88.1%). Almost half of those with known monthly family income had income below 900 Euros. The majority of the parents were employed (83.2% of the fathers and 65.5% of the mothers), married or in cohabitation (84.2%), had less than 12 years of education (71.3% of the fathers and 69.0% of the mothers) and their child had insurance (88%). From this crude univariable analysis, national origin, family income, possessing insurance, parents’ education level, and area of residence seem to be associated with parents’ attitudes towards vaccination.

**Table 2:** Characteristics of 447 study participants and their households overall and by attitude and skepticism status towards vaccination. *Weighted median (IQR), **: Chi-square test was performed, ***: Wilcoxon test was performed. Weighted percentages and median (IQR) are presented. The data has been represented as N (%) for categorical variables and median (IQR) for continuous variables. The level of significance was set at p-value<0.05. IQR: Interquartile range.

	Total	Attitude towards vaccination				Skepticism status towards vaccination		
		Absolutely positive	Positive	Negative	p-value	No	Yes	p-value
	n=447	n=74	n=341	n=32		n=259	n=188	
	N (%)	N (%)	N (%)	N (%)		N (%)	N (%)	
Questionnaire completed by								
Mother	356 (68.0)	60 (17.4)	272 (76.1)	24 (6.5)	0.70**	208 (58.7)	148 (41.3)	0.44**
Father	91 (32.0)	14 (16.0)	69 (74.8)	8 (9.2)		51 (54)	40 (46)	
Age* (years)								
	42 (36.2, 48.0)	42 (35.0, 50.4)	41.82 (36.59, 48.0)	43.11 (37.0, 50.0)	0.84*	41.39 (36.0, 48.0)	42.62 (36.1, 48.9)	0.34*
National origin								
Greek	394 (88.1)	56 (14.8)	309 (77.8)	29 (7.4)	0.02**	221 (54.7)	173 (45.3)	<0.01**
Other	51 (11.5)	18 (34.3)	31 (59.8)	2 (5.9)		38 (77.8)	13 (22.2)	
Unknown	2 (0.4)	0 (0)	1 (60.1)	1 (39.9)		0 (0)	2 (100.0)	
Family monthly Income (Euros)								
<900	159 (35.8)	31 (20.8)	116 (72.3)	12 (6.9)	0.03**	95 (60.1)	64 (39.9)	0.44**
900-1700	120 (26.0)	14 (11.5)	92 (76.6)	14 (11.9)		67 (52.1)	53 (47.9)	
>1700	76 (18.9)	10 (10.3)	64 (86.8)	2 (2.9)		41 (54.1)	35 (45.9)	
Unknown	92 (19.3)	19 (23.9)	69 (69.6)	4 (6.5)		56 (61.6)	36 (38.4)	
Father’s employment status								
Employed	373 (83.2)	57 (15.6)	288 (76.9)	28 (7.6 )	0.34**	215 (56.2)	158 (43.8)	0.27**
Unemployed	60 (13.8)	14 (24.4)	44 (70.3)	2 (5.3)		37 (64.2)	23 (35.8)	
Unknown	14 (3.0)	3 (21.9)	9 (65.9)	2 (12.2)		7 (53.4)	7 (46.6)	
Mother’s employment status								
Employed	298 (65.5)	48 (16.1)	231 (77.0)	19 (6.9)	0.75**	177 (56.9)	121 (43.1)	0.72**
Unemployed	142 (32.7)	25 (18.4)	105 (73.4)	12 (8.2)		79 (58.8)	63 (41.2)	
Unknown	7 (1.8)	1 (23.6)	5 (66.0)	1 (10.4)		3 (38.7)	4 (61.3)	
Insurance								
Yes	398 (88.2)	59 (14.9)	311 (77.9)	28 (7.2)	0.04**	227 (55.8)	171 (4.2)	0.19**
No	43 (10.4)	12 (31.0)	27 (59.0)	4 (10.0)		27 (66.9)	16 (33.1)	
Unknown	6 (1.4)	3 (45.1)	3 (54.9)	0 (0.0)		5 (70.9)	1 (29.1)	
Marital status								
Married/cohabitation	377 (84.2)	58 (15.8)	293 (77.4)	26 (6.8)	0.21**	227 (59.2)	150 (40.8)	0.07**
Single	70 (15.8)	16 (23.5)	48 (66.2)	6 (10.3)		32 (46.7)	38 (53.3)	
Number of children								
1	125 (29.7)	22 (16.6)	94 (75.6)	9 (7.8)	0.91**	69 (55.2)	56 (44.8)	0.85**
2	203 (43.8)	31 (17.2)	157 (75.2)	15 (7.6)		117 (56.1)	86 (43.9)	
3	79 (17.5)	15 (16.2)	56 (73.9)	8 (9.9)		47 (58.3)	32 (41.7)	
4 or more	22 (5.1)	4 (20.0)	18 (80.0)	0 (0.0)		14 (65.7)	8 (34.3)	
Unknown	18 (3.9)	2 (17.0)	16 (83.0)	0 (0.0)		12 (67.7)	6 (32.3)	
Father’s education (years)								
≤12	332 (71.3)	61 (19.2)	254 (75.8)	17 (5.0)	0.01**	197 (58.9)	135 (41.1)	0.49**
> 12	108 (27.2)	12 (11.0)	83 (76.4)	13 (12.6)		61 (54.8)	47 (45.2)	
Unknown	7 (1.5)	1 (19.1)	4 (56.2)	2 (24.7)		1 (19.1)	6 (80.9)	
Mother’s education (years)								
≤12	316 (69)	55 (18.2)	237 (73.9)	24 (7.9)	0.42**	184 (58.2)	132 (41.8)	0.78**
>12	123 (28.9)	17 (13)	98 (80.2)	8 (6.8)		72 (56.6)	51 (43.4)	
Unknown	8 (2.1)	2 (30.9)	6 (69.1)	0 (0.0)		3 (33.5)	5 (66.5)	
Area of residence								
Urban	272 (66.4)	50 (18.5)	203 (74.5)	19 (7.0)	0.01**	151 (54.7)	121 (45.3)	0.3**
Semi-urban	86 (18.0)	5 (4.4)	74 (85.6)	7 (10.0)		51 (59.7)	35 (40.3)	
Rural	89 (15.6)	19 (24.9)	64 (69.1)	6 (6.0)		57 (64.6)	32 (35.4)	

In Table 3, results from multivariable multinomial logistic regression analysis assessing factors associated with parents’ attitudes towards vaccinations are presented. Compared to the base category of parents having absolute positive attitudes towards vaccination, those of Greek origin were significantly more likely to have positive (adjusted relative risk ratio (aRRR):3.35; 95% CI: 1.53-7.30) attitudes than those of non-Greek origin. Similarly, higher parents’ education was associated with being more likely to have negative attitudes rather than absolute positive attitudes (aRRR:2.98; 95% CI: 1.05-8.44). Interestingly, compared to the base category of having absolute positive attitudes, parents living in semi-urban areas were much more likely to have positive (aRRR:4.84; 95% CI: 1.77-13.29) or even negative attitudes (aRRR:6.43; 95% CI: 1.69-24.56) towards vaccination compared to those living in urban areas whereas those living in rural areas had similar attitudes to those living in urban areas. None of the other investigated characteristics were statistically significantly associated with attitudes toward vaccination. 

**Table 3:** Adjusted relative risk ratios (aRRRs) and associated 95% confidence intervals (95% CI) derived from the final multivariable multinomial logistic regression assessing factors associated with parents’ attitudes towards vaccination. *: Higher among parents. Base category: Absolutely positive attitude. Results are shown after imputing missing data by multiple imputations. The level of significance was set at p-value<0.05.

	Fully adjusted model							
	Positive vs absolutely positive			Negative vs absolutely positive				
	aRRR	95% CI	p-value	aRRR		95% CI	p-value	
Interviewed parent: Mother (ref)								
Father	1.03	0.52 - 2.04	0.94	1.35	0.48 - 3.81			0.57
Age (years)	0.97	0.94 - 1.01	0.08	0.97	0.93 - 1.02			0.23
National Origin: Non-Greek (ref)								
Greek	3.35	1.53 - 7.30	0.002	2.84	0.58 - 13.84			0.20
Educational level^*^: ≤ 12 years (ref)								
> 12 years	1.45	0.77 - 2.76	0.25	2.98	1.05 - 8.44			0.04
Area of residence: Urban (ref)								
Semi-urban	4.84	1.77 - 13.29	0.002	6.43	1.69 - 24.56			0.006
Rural	0.79	0.40 - 1.55	0.49	0.89	0.27 - 2.96			0.85

Focusing on factors associated with being skeptical towards vaccination, results from the final multivariable logistic model (Table 4) showed that, among all investigated factors, only national origin and being married/in cohabitation were significantly associated with being skeptical. Those of Greek origin, in line with the results for parents’ attitudes, had almost three times higher odds of being skeptical compared to those of non-Greek origin (adjusted odds ratio (aOR):2.86; 95% CI: 1.36-5.98) whereas married/in cohabitation parents had 40% lower odds of being skeptical compared to single parents (aOR:0.60; 95% CI: 0.35-1.06), the association being though only marginally significant.

**Table 4:** Adjusted odds ratios (aORs) and associated 95% confidence intervals (95% CI) along corresponding p-values derived from the final multivariable logistic model for factors associated with being skeptical towards vaccination. *: Non-skeptical is the reference category. Results are shown after imputing missing data by multiple imputations. The level of significance was set at p-value<0.05.

	Fully adjusted model		
	aOR*	95% CI	p-value
Interviewed parent: Mother (ref)			
Father	1.23	0.76 - 2.00	0.40
Age (years)	1	0.98 - 1.02	0.85
Nationality: non-Greek (ref)			
Greek	2.86	1.36 - 5.98	0.005
Marital status: Single (ref)			
Married/cohabitation	0.60	0.35 - 1.03	0.06

## Discussion

In this sample of parents living in Greece, the great majority (92.9%) had a positive or an absolutely positive attitude towards vaccinations and 8% had a negative attitude. Nevertheless, a substantial proportion of the parents, reaching 42%, were skeptical about vaccinations, although most of this group agreed on the necessity of vaccines for their child’s health. Parents with higher educational levels and of Greek origin were more likely to have a less positive or negative attitude towards vaccinations as did those living in semi-urban areas. Similarly, being skeptical was more likely among parents of Greek origin and among singles. Age, gender, employment, and income were not associated with their attitude, positive or negative, or skepticism status toward vaccinations, in this study population, which included participants across all of Greece. 

The results of this study in terms of the parents’ attitudes towards vaccinations are not directly comparable with those of previous relevant national or multinational studies with participants from Greece, due to differences in the study methodology and definitions, population sampling and origin, as well as tools used to measure attitudes towards vaccinations [9,11,13-15]. Nevertheless, in a national population-based survey using a representative sample of six-year-old students and their parents/guardians during 2004-2005, which implemented the same tool, the percentage of parents who agreed that vaccinations are necessary was larger than in this study, reaching almost 100% [13]. Also, the percentage of parents who feared that vaccines may be harmful, and those who preferred natural immunity over the immunity induced by vaccines, was much lower in 2004-2005, 3.3% and 19.4% respectively, compared to this study. However, a recently conducted online study focusing on mothers’ attitudes and perceptions about children's vaccination in Greece reported that most participants (92%) were supporters of vaccination as a useful and safe practice against serious infections [16]. This result is broadly in line with our result, but skepticism was not reported in this study; additionally, due to its methodology (online survey), it recruited mothers who were younger, of higher education, and of higher income. Thus, the results are not directly compared to ours. 

In a survey conducted in 2018 among 28 EU member states, 92.8% of the participants from Greece agreed that vaccines are important for children, and 84.5% that vaccines are safe [11]. In another relevant study conducted in 18 European countries, parents with at least one child one to four years old, replied to a web-based questionnaire provided by primary care pediatricians [15]. Most of the parents (91%) thought that vaccines are important for children, whereas among the 210 parents from Greece, 33% were hesitant and 57% were not at all hesitant towards vaccinations.

The association of higher educational level with a less positive or even negative attitude towards vaccinations, as well as with skepticism towards vaccinations, has been reported in previous studies both in Greece and abroad [17,18]. It has been postulated that parents of higher educational levels may be more hesitant or critical towards immunizations due to their increased access to various sources of information, including those promoting extreme, false, or misleading information and opinions, that may increase their concerns [17,18]. It is also interesting that high health literacy, defined as the knowledge and skills required to make health decisions, which correlates with a high educational level, has been associated with higher rates of vaccine rejection and hesitation in some studies [19]. On the other hand, maternal or parental higher education has been consistently associated with better vaccine acceptance and uptake in different areas of the world, mainly in low and middle-income countries [15,20,21]. In these countries, education may play a more important role than in high-income countries since it is associated with improved access to the healthcare system and enhanced cognition and communication skills that encourage healthier lifestyle choices and vaccine uptake [20-22]. Additionally, the online survey conducted by Fakonti et al. linked the higher educational level of Greek mothers to increased support for vaccination practices [16]. Overall, it has been pointed out that education can act in both directions, either as a barrier or as a promoter of vaccine confidence, depending on the context, setting, or vaccine, concluding that there is no clear pattern between the two and that being educated does not necessarily imply confidence to vaccination [4,10,23].

Greek nationality was associated with less positive attitudes and more skepticism towards vaccination compared to non-Greek in this study. It is interesting that in studies where vaccine uptake is measured, although not identical to the concept of attitudes/perception about vaccinations, the opposite has been found since Greek nationality was associated with higher vaccine uptake compared to non-Greek, a finding that has been partly attributed to relatively decreased access to preventive healthcare services and different cultural beliefs of the non-Greek parents [5,13,14,18]. 

Gender is recognized as one of the determinants of vaccine hesitancy [4]. Although not consistently, women have been found to be more confident towards vaccinations compared to men [24]. With respect to age, younger age groups usually express less often positive views towards vaccinations compared to the older ones [11], although opposite findings have also been found, and seem to depend on the specific vaccine [11,24]. None of these factors were statistically significantly associated with parent’s attitudes and perceptions in this study sample. Being married or cohabiting has been associated with parental attitudes of higher vaccine uptake [14,16]. In our study, marital status was not significantly associated with attitudes toward vaccination, but single parents tend to be more skeptical.

Furthermore, we found that residing in semi-urban areas was associated with having less positive attitudes towards immunizations compared to residing in urban areas. This finding is not easily interpretable based on the available data, as residing in semi-urban areas, most probably, has intermediate characteristics between those of urban and rural areas of residence. Previous studies have shown differences in vaccine hesitancy and uptake between residents of urban and rural areas, especially in relation to COVID-19 vaccines [25-27]. Although associations found are not consistent, residence in rural areas has been associated more often been associated with higher vaccine hesitancy and lower vaccination rates [25]. Attempts to explain these differences point to disparities in economic, cultural, and educational barriers between urban and rural areas as well as structural barriers related to access to preventive care services in the case of vaccine uptake.

An inherent limitation of our study is its cross-sectional design, which generally precludes the establishment of causal inferences. However, the nature of some of the socio-demographic factors involved does not change over time rendering the traditional concern over causal inference less pertinent in the context of this study. The questionnaire used to measure attitudes towards immunizations has been used before in the context of Greek national immunization coverage surveys and has been validated in a sample of parents living in Greece, but it is not directly comparable with similar data from other countries [13]. Also, the questionnaire did not contain questions to address attitudes towards a particular vaccine or vaccines thus we were not able to reveal whether reported attitudes were related to a specific vaccine or vaccines and not to vaccines in general. Underreporting of negative perceptions about vaccinations is also possible since hesitant or negative parents towards immunizations may not have agreed to participate in the study, or if they have agreed, they might have provided more socially desirable replies. Another limitation is that we could not link the vaccination attitudes with the actual vaccination uptake. Lastly, although EMENO, and thus also this sub-study, was a nationwide health examination survey based on a representative sample of adults living in Greece, as not all eligible EMENO participants consented to participate in this sub-study, our findings could not be generalizable to the whole population of parents living in Greece. In particular, the rural residents were under-represented and urban residents were over-represented in our sample. Also, as those of higher education were more likely to participate in this sub-study and higher education is associated with less positive attitudes towards vaccines, our estimate of the proportion of adults with unfavorable attitudes to vaccinations may be, to some degree, overestimated. However, in our analysis, we attempted to adjust for such imbalances in response rates by applying inverse probability weighting. 

This study highlights the positive attitudes of Greek parents towards vaccination but also the skepticism, that, at the same time, characterizes them. This last finding may lead to a significant barrier to childhood vaccination, especially after the COVID-19 pandemic. The global impact of the COVID-19 pandemic has exacerbated this situation, leading to a significant decline in routine childhood immunizations worldwide due to disruptions in the delivery and uptake of immunization services [28]. Additionally, heightened public concerns about side effects related to COVID-19 vaccines have further raised questions and increased hesitancy towards vaccines in general, with an anticipated upward trajectory in vaccine hesitancy [29]. Last but not least, parents’ views on the long-term risk of the COVID-19 vaccine and their perceived responsibility for their children’s health were linked to lower vaccine uptake among vaccinated parents’ children [30]. However, a recently published online cross-sectional study regarding Greek mothers did not provide evidence that COVID-19 antivaccination campaigns have changed significantly Greek mothers’ attitudes and perceptions toward childhood vaccination overall [16].

In conclusion, the attitudes towards vaccinations among parents in Greece, as observed in this sample, were predominantly positive, reflecting an appreciation for the importance of vaccinations and adherence to scientific recommendations. However, a noteworthy proportion of parents exhibited skepticism, and a minority held negative views, potentially impacting the overall childhood vaccination coverage in the country. Given these circumstances, there is an urgent need to intensify coordinated and multi-level efforts to effectively address the declining confidence and acceptance of vaccines among parents.

## Conclusions

The majority of parents in this sample of parents living in Greece held positive attitudes towards vaccinations. This indicates that they recognized the significance of immunizations and followed scientific recommendations. Nevertheless, a significant part of parents were skeptical, and a minority maintained unfavorable opinions, which could potentially affect the total rate of children immunization in the country.
